# Cyclosporine Biosynthesis in *Tolypocladium inflatum* Benefits Fungal Adaptation to the Environment

**DOI:** 10.1128/mBio.01211-18

**Published:** 2018-10-02

**Authors:** Xiuqing Yang, Peng Feng, Ying Yin, Kathryn Bushley, Joseph W. Spatafora, Chengshu Wang

**Affiliations:** a Key Laboratory of Insect Developmental and Evolutionary Biology, CAS Center for Excellence in Molecular Plant Sciences, Shanghai Institute of Plant Physiology and Ecology, Chinese Academy of Sciences, Shanghai, China; b University of Chinese Academy of Sciences, Beijing, China; c Department of Plant Biology, University of Minnesota, St. Paul, Minnesota, USA; d Department of Botany and Plant Pathology, Oregon State University, Corvallis, Oregon, USA; e School of Life Science and Technology, ShanghaiTech University, Shanghai, China; Cornell University

**Keywords:** cyclosporine, *Tolypocladium inflatum*, antifungal activity, biosynthetic pathway, virulence

## Abstract

The cycloundecapeptide cyclosporin A (CsA) was first isolated from the insect-pathogenic fungus *Tolypocladium inflatum* for its antifungal activity and later developed as an immunosuppressant drug. However, the full biosynthetic mechanism of CsA remains unknown and has puzzled researchers for decades. In this study, the biosynthetic gene cluster is suggested to include 12 genes encoding enzymes, including the nonribosomal peptide synthetase (NRPS) (SimA) responsible for assembling the 11 amino acid substrates of cyclosporine and a polyketide synthase (PKS) (SimG) to mediate the production of the unusual amino acid (4*R*)-4-[(*E*)-2-butenyl]-4-methyl-l-threonine (Bmt). Individual deletion of 10 genes, isolation of intermediates, and substrate feeding experiments show that Bmt is biosynthesized by three enzymes, including SimG, SimI, and SimJ. The substrate d-alanine is catalyzed from l-alanine by alanine racemase SimB. Gene cluster transcription is regulated by a putative basic leucine zipper (bZIP)-type protein encoded by the cluster gene *SimL*. We also found that the cluster cyclophilin (SimC) and transporter (SimD) genes contribute to the tolerance of CsA in the CsA-producing fungus. We also found that cyclosporine production could enable the fungus to outcompete other fungi during cocultivation tests. Deletion of the CsA biosynthetic genes also impaired fungal virulence against insect hosts. Taking all the data together, in addition to proposing a biosynthetic pathway of cyclosporines, the results of this study suggest that CsA produced by this fungus might play important ecological roles in fungal environment interactions.

## Abstract (precis)

The cyclopeptide cyclosporin A was first isolated from the filamentous fungus *Tolypocladium inflatum* showing antifungal activity and was later developed as an immunosuppressant drug. We report the biosynthetic mechanism of cyclosporines that are mediated by a cluster of genes encoding NRPS and PKS controlled by a bZIP-type transcriptional regulator. The two unusual amino acids Bmt and d-Ala are produced by the PKS pathway and alanine racemase, respectively. The cyclophilin and transporter genes jointly contribute to fungal self-protection against cyclosporines. Cyclosporine confers on *T. inflatum* the abilities to outcompete other fungi in competitive interactions and to facilitate fungal infection of insect hosts, which therefore benefits fungal adaptations to different environments.

## INTRODUCTION

Cyclosporine (CSN) was first isolated and structurally identified as a cyclic undecapeptide metabolite from the insect-pathogenic and soil-dwelling fungus *Tolypocladium inflatum* in the early 1970s (1, 2). Cyclosporin A (CsA) was developed and approved as an immunosuppressant drug used in organ transplantation in 1983, and more than 30 analogs of CsA have been identified with different biological activities, including immunosuppressive, antifungal, antiviral, and antiparasitic properties (3).

Due to the high value of CsA, scientists have been attempting to dissect its biosynthetic mechanism for decades. In the early 1990s, the nonribosomal peptide synthetase (NRPS) gene (termed *SimA*) was cloned, and its role in CSN biosynthesis was functionally confirmed (4, 5). However, the biosynthetic pathway of CSN is still unclear. CsA is composed of 11 amino acids, including two nonproteinogenic substrates d-alanine (d-Ala) and (4*R*)-4-[(E)-2-butenyl]-4-methyl-l-threonine (Bmt). An alanine racemase (AlaR) with a high activity in conversion of l-Ala to d-Ala was previously purified from *T. inflatum* and shown to have a role in CSN biosynthesis (6, 7). Bmt has been characterized biochemically and proposed to be produced by a polyketide synthase (PKS) (8–10). On the basis of different methods of prediction and gene expression analysis, 10, 14, or 22 genes have been proposed for the CsA biosynthetic gene cluster (1). The functions of these genes have not been investigated until now.

CsA was first characterized as an antifungal compound (3). Thus, CsA production can be toxic to the producing fungus *T. inflatum*. The target of CsA was first determined as cyclophilin A (CypA) which exhibits a peptidyl-prolyl isomerase activity (11). Multiple cyclophilin genes are present in different organisms and have been found to play diverse biological roles, including stress tolerance, signal transduction, gene regulation, and pathogenesis (12). For example, 11 cyclophilin genes were characterized to alternatively mediate fungal conidiation, heat tolerance, virulence, and sensitivity/resistance to CsA in the insect pathogen Beauveria bassiana, a close relative of *T. inflatum* (13). It was hypothesized that the CsA binding feature of cyclophilin could potentiate the tolerance of *T. inflatum* cells against the CSN products (3, 14). It was found that a cyclophilin gene within the CsA biosynthetic gene cluster was highly expressed by the fungus in both a CSN induction medium and a medium containing insect hemolymph (1). However, the protective effect of this gene on CsA cytotoxicity has not been verified. It is unknown whether there is any other gene (e.g., the membrane transporter) that also contributes to self-tolerance/detoxification of CsA. The CSN producer *T. inflatum* is a pathogen of beetles (1). It has been reported that CsA could inhibit insect immune responses by targeting lipophorins, the regulators of both humoral and cellular immune responses in insects (15, 16). The contribution of CSNs to fungal virulence remains to be determined.

In this study, using the predicted biosynthetic gene cluster from the genome sequence, we performed multiple gene deletions and intermediate isolation and substrate feeding experiments to dissect the CsA biosynthetic mechanism in *T. inflatum*. We also explored the possible adaptive functions of CSNs to the CSN-producing fungus by conducting fungal competition tests and insect bioassays. We find that CSNs allow the producing fungus to outcompete other fungi and facilitates fungal infection of insect hosts.

## RESULTS

### Functional verification of the clustered genes.

**FIG 1:**
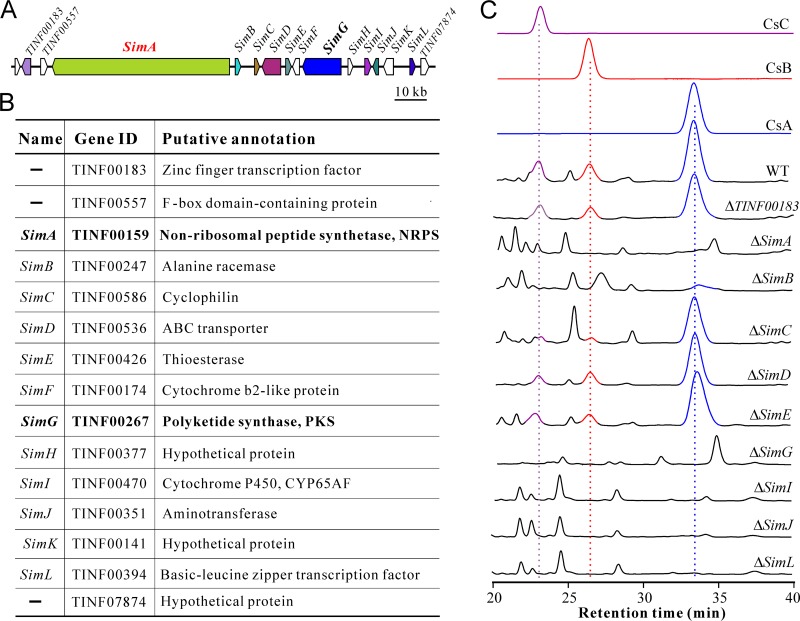
Prediction and functional verification of the CSN biosynthetic gene cluster. (A) Schematic map of the biosynthetic gene cluster. The genes are named following the previously designated *SimA* gene for the core NRPS gene. (B) Annotation of the gene contents within the gene cluster. ID, identifier. (C) Loss-of-function verification of the contributions of different genes to CSN biosynthesis. HPLC analysis of CSN production by the WT and different null mutants of *T. inflatum*. The standards CsA, CsB, and CsC were included in parallel analysis.

On the basis of our previous predictions (1), 15 genes were investigated, and 12 of the genes were named *SimA* to *SimL* for the putative biosynthetic gene cluster of CSNs (Fig. 1A). This gene cluster is predicted to encode the enzymes NRPS (SimA) and PKS (SimG) as well as the alanine racemase (SimB), cyclophilin (SimC), ABC transporter (SimD), cytochrome P450 (SimI), aminotransferase (SimJ), and transcription factor (TF) (SimL). Additional analysis of the previously annotated gene *TINF00195* suggested that it is unlikely to encode a functional dehydrogenase, as its open reading frame is only 198 nucleotides. *TINF00195* was not further examined. The hypothetical protein genes *SimF*, *SimH*, and *SimK* were also excluded in further analysis due to their unclear functional contributions to CSN biosynthesis. SimE (previously predicted to be an esterase-like protein) contains a putative thioesterase (TE) domain (Pfam00975; 1.13e−07) (Fig. 1B). To determine the boundary of the gene cluster and the contributions of these genes to CSN biosynthesis, 10 genes were individually deleted by homologous recombination via *Agrobacterium*-mediated transformation of the wild-type (WT) strain of *T. inflatum* (see Fig. S1 in the supplemental material), including a putative zinc finger TF *TINF00183* gene upstream of *SimA*. After growth in the CSN induction medium containing fructose (fructose CSN induction medium) (17), high-performance liquid chromatography (HPLC) analysis demonstrated that the WT strain could produce CsA, cyclosporin B (CsB), and cyclosporin C (CsC) during chromatographic profiling with these standards. Extracts of mutants lacking either *SimA*, *SimG*, *SimI*, *SimJ*, or *SimL* resulted in the failure to detect CsA to CsC (Fig. 1C). Disruption of the putative racemase gene *SimB* impaired but did not completely abolish fungal ability to produce CsA. No obvious differences were observed between WT and null mutants of *SimC*, *SimD*, *SimE*, or *TINF00183* in CSN production.

10.1128/mBio.01211-18.1 FIG S1 Target gene deletion and PCR verification. (A) Schematic map for gene deletion. The 5′- and 3′-flanking regions of the target gene were amplified with primer pairs U1/L1 and U2/L2. The products were then cloned into the binary vector containing the drug resistance gene for transformation of the wild-type strain. Primers F (F stands for forward) and R (R stands for reverse) designed for each target gene are used for PCR verification. (B) PCR verification of the drug-resistant mutants with or without the wild-type (WT) strain as a reference. Ect, ectopic transformant resulting in two PCR bands where the smaller band in each panel is from the WT gene. The Δ*TINF06009* (Δ*06009*) mutant was obtained in the Δ*SimB* background for double deletions of these two genes. Download FIG S1, TIF file, 0.83 MB. Copyright © 2018 Yang et al. 2018 Yang et al. This content is distributed under the terms of the Creative Commons Attribution 4.0 International license.

### Conversion of l-Ala to substrate d-Ala.

**FIG 2:**
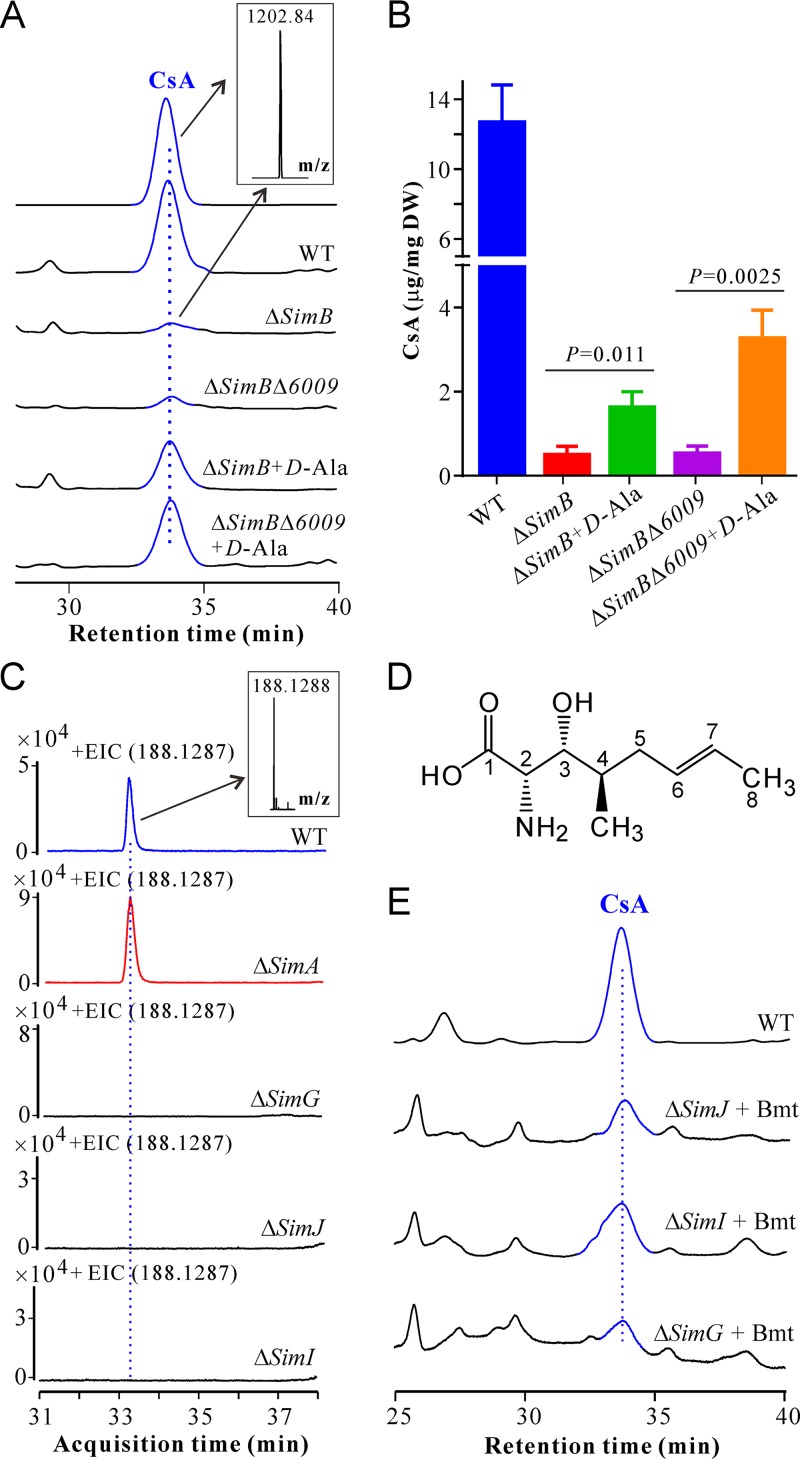
Verification of the genes involved in d-Ala conversion and Bmt biosynthesis. (A) HPLC analysis of CsA production by WT and different mutants with or without the addition of d-Ala. The inset shows the mass spectra detected for the CsA and Δ*simB* samples. *m/z*, [M+H]^+^; Δ*SimB*Δ*6009*, Δ*SimB* Δ*TINF06009* double mutant. (B) Quantification analysis of CsA production by WT and different mutants. The strains were grown in fructose CSN induction medium with or without the supplementation of d-Ala (at a final concentration of 20 mM) for 10 days. The mycelia were then harvested for CSN extraction. Values are means plus standard errors (SE) (error bars). DW, mycelium dry weight. (C) LC-MS analysis of the extracted ion chromatography (EIC) showing the production or nonproduction of Bmt by WT and mutant strains. *m/z*, [M+H]^+^. (D) Chemical structure of Bmt. (E) Supplementation of Bmt (at a final concentration of 85 μM) in the growth medium enabled the null mutants to produce CsA (peaks shown in blue).

Previously, it was reported that the putative racemase encoded by *SimB* (referred to originally as *AlaR*; GenBank accession no. A40406) was able to convert l-Ala to d*-*Ala (7). We found that deletion of *SimB* significantly reduced but did not completely abolish CSN production in *T. inflatum* (Fig. 1C). It was previously noted that synthesis of d*-*Ala could also be mediated by a threonine aldolase (7). Analysis of the *T. inflatum* genome sequence identified a putative threonine aldolase (TINF06009; sharing 43% identity with SimB at the amino acid level), located outside the putative CsA biosynthetic gene cluster. To verify the potential contribution of this gene, deletion of *TINF06009* was conducted in the Δ*SimB* background for a double deletion of these two genes. HPLC analysis revealed that trace amounts of CsA could still be produced by the double mutant (Δ*SimB* Δ*TINF06009* [Δ*SimB*Δ*6009*]) (Fig. 2A). We also performed feeding assays and found that supplementation of d*-*Ala could significantly increase the cellular accumulation of CsA by both Δ*SimB* (*P = *0.011) and Δ*SimB*Δ*6009* (*P = *0.0025) mutants compared with the corresponding null mutants growing in the medium without d*-*Ala. Unexpectedly, feeding with d*-*Ala could not fully enable the mutants to produce the same amount of CsA as the WT did, for unknown reasons (Fig. 2B).

### Bmt biosynthesis.

We found that deletion of the PKS gene *SimG* abolished the fungal ability to produce CSNs (Fig. 1C). It is hypothesized that a PKS could be responsible for the biosynthesis of the Bmt with a deduced structure 3*R*-hydroxyl-4*R*-methyl-6*E*-octenoic acid (termed compound b1) (9). We performed liquid chromatography (LC)-mass spectrometry (MS) analysis of both the WT and mutant samples in order to detect Bmt or other similar carboxylic acid type intermediates. It was found that, relative to the WT, Δ*SimG*, Δ*SimJ*, and Δ*SimI* mutants lost the abilities to produce a compound with a [M+H]^+^ molecular ion of 188.1288, which was more highly accumulated in the Δ*SimA* mutant than in the WT (Fig. 2C). This chemical was then purified based on its mass through two rounds of preparative LC-MS analysis, and the obtained compound was subjected to one-dimensional (1D) and two-dimensional (2D) nuclear magnetic resonance (NMR) analysis (Fig. S2 and S3). The data obtained revealed that this compound is Bmt (Fig. 2D; see also Table S1 in the supplemental material). Unfortunately, efforts to isolate the predicted Bmt precursors b1 to b3 were not successful, given that the isoabsorbance plot profiles were different for the mutants in HPLC analysis equipped with a diode array detector (DAD) (Fig. S4). We also conducted substrate feeding assays with the purified Bmt and found that the supplementation of Bmt could restore the ability of all three mutants, Δ*SimG*, Δ*SimJ*, and Δ*SimI* mutants, to produce CsA (Fig. 2E). Thus, taken together, Bmt is likely the product of the following biochemical pathway: SimG to SimI to SimJ, an intermediate in the production of CsA.

10.1128/mBio.01211-18.2 FIG S2 Bmt structure analysis. (A) 1D NMR analysis of Bmt in D_2_O. (B) ^1^H-^1^H correlation spectroscopy (COSY) analysis of Bmt in D_2_O. Download FIG S2, TIF file, 1.14 MB. Copyright © 2018 Yang et al. 2018 Yang et al. This content is distributed under the terms of the Creative Commons Attribution 4.0 International license.

10.1128/mBio.01211-18.3 FIG S3 2D NMR analysis of Bmt in D_2_O. HMBC, heteronuclear multiple bond correlation; HSQC, heteronuclear singular quantum correlation. Download FIG S3, TIF file, 1.17 MB. Copyright © 2018 Yang et al. 2018 Yang et al. This content is distributed under the terms of the Creative Commons Attribution 4.0 International license.

10.1128/mBio.01211-18.4 FIG S4 Isoabsorbance plots of the CsA standard, WT, and selected mutant strains. The data were generated from the HPLC diode array detector. Download FIG S4, TIF file, 1.91 MB. Copyright © 2018 Yang et al. 2018 Yang et al. This content is distributed under the terms of the Creative Commons Attribution 4.0 International license.

10.1128/mBio.01211-18.9 TABLE S1 NMR data for identification of Bmt. Download Table S1, PDF file, 0.07 MB. Copyright © 2018 Yang et al. 2018 Yang et al. This content is distributed under the terms of the Creative Commons Attribution 4.0 International license.

### Pathway-specific regulation control.

**FIG 3:**
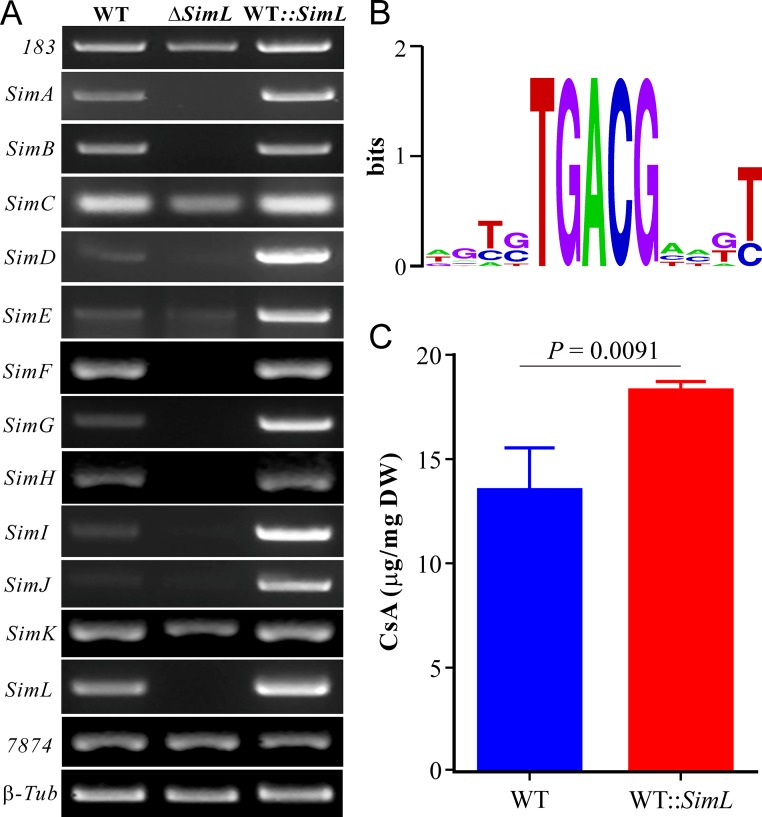
Functional verification of the pathway-specific transcription factor SimL. (A) RT-PCR analysis of gene expression. The WT, Δ*SimL*, and WT::*SimL* strains were grown in fructose CSN induction medium for 10 days, and the mycelia were harvested for RNA extraction and gene expression analysis. *TINF00183* and *TINF07874* are indicated as *183* and *7874*, respectively. β-*Tub*, β-Tubulin. (B) *In silico* analysis of the putative binding motif by the bZIP-type TF SimL. (C) Comparative quantification of CsA production. The WT and WT::*SimL* strains were grown in fructose CSN induction medium for 10 days, and the mycelia were harvested for cyclosporine extraction. There were three replicates for each sample. Values are means plus SE.

Fungal secondary metabolism is controlled by both global regulator(s) and/or pathway-specific TFs (18, 19). There are two TFs (i.e., *TINF00183* and *SimL*) in close proximity to the CsA gene cluster. Our previous transcriptome sequencing (RNA-seq) analysis indicated that the expression of *TINF00183* was not upregulated in an inductive medium (1). To explore a possible role in CsA biosynthesis, deletion mutants of these putative TFs was generated. Analysis of mutant extracts revealed that the basic leucine zipper (bZIP)-type TF SimL, but not the zinc finger TF TINF00183, controlled CSN production (Fig. 1C). To further confirm the function of SimL, we performed gene overexpression. Thus, *SimL* was put under the control of the constitutive glyceraldehyde-3-phosphate dehydrogenase (GpdA) gene (*TINF02918*) promoter, and the cassette was used to transform the WT strain of *T. inflatum*. Three independent transformants were selected for trial evaluations of CsA production, and the one with the highest yield was used for further analysis. After the fungi were grown in the fructose CSN induction medium for 10 days, RNAs were extracted for semiquantitative reverse transcription-PCR (RT-PCR) analysis. Consistent with the results of our previous RNA-seq analysis (1), the results indicated that the clustered genes were co-upregulated, with the cyclophilin gene *SimC* being the most highly transcribed by the fungus (Fig. 3A). Except for *SimC*, *SimK*, *TINF00183*, and *TINF07874*, deletion of *SimL* deactivated the expression of other genes. In contrast, overexpression of *SimL* highly induced the transcription of the cluster genes compared with the WT strain (Fig. 3A).

The characteristic binding motif of fungal bZIP-type TFs is TGACG (20). *In silico* analysis identified the presence of this conserved motif in the promoter regions of the clustered genes (Fig. 3B). Quantification analysis of CsA production also revealed that *SimL* overexpression significantly (*P = *0.0091) increases the yield of CsA production (26.15%) (Fig. 3C). Taken together, the data indicate that SimL is responsible for the pathway-specific control of CSN biosynthesis in *T. inflatum*.

### Deduction of the biosynthetic pathway.

**FIG 4:**
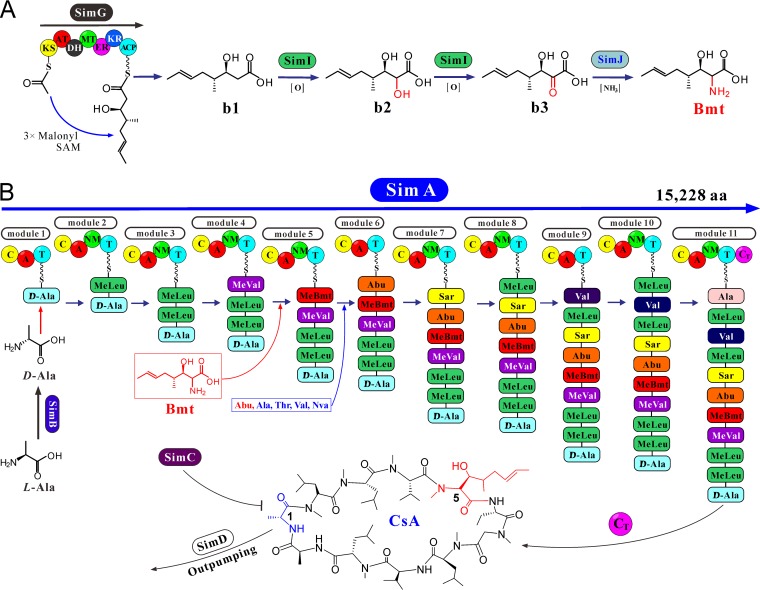
Proposed pathway for CsA biosynthesis. (A) Bmt biosynthesis by the PKS pathway. The PKS SimG domains include the following: β-ketoacyl synthase (KS), acyltransferase (AT), dehydrogenase (DH), methyltransferase (MT), enoylreductase (ER), ketoreductase (KR), acyl carrier protein (ACP), *S*-adenosylmethionine (SAM). The chemical structure of compounds b1 to b3 and Bmt are shown. (B) Schematic structure of NRPS SimA and the machinery of CsA biosynthesis. There are 11 modules of SimA, and each module contains the condensation (C), adenylation (A), thiolation (T), and/or *N-*methylation (NM) domains. The terminal C domain (C_T_) is implicated in cyclization of the peptidyl chains to form CsA and its analogs. The cyclophilin SimC and exporter SimD may jointly contribute to cell tolerance of CSNs. Abu, aminobutyric acid; Sar, sarcocine; Nva, norvaline; MeLeu, methylleucine; aa, amino acids.

Having determined functions of several genes in the cluster, we next tried to deduce the CsA biosynthetic pathway. As indicated above, SimG, SimI, and SimJ are all required for the production of Bmt (Fig. 1C and Fig. 2E). It is likely that the PKS SimG mediates the biosynthesis of compound b1 from acetyl coenzyme A (acetyl-CoA), malonyl-CoA, and *S*-adenosylmethionine, and compound b1 will then be repeatedly oxidized by SimI to 3*R*-hydroxy-4*R*-methyl-2-keto-6*E*-octenoic acid (compound b3). The latter is likely converted to Bmt through the action of the aminotransferase SimJ (Fig. 4A).

Analysis of SimA protein indicated that the NRPS contains 11 modules responsible for sequential uptake of substrates and chain elongation. In addition to the core condensation-adenylation-thiolation (C-A-T) domains present in each module, three modules (modules 1, 6, and 9) contain an additional *N*-methylation (NM) domain (Fig. 4B). Bmt has been considered to be the first substrate for CsA biosynthesis (3, 6). Since Bmt is methylated in CSNs and the first module of SimA does not contain a NM domain, it appears unlikely that the first module of SimA is responsible for the addition of Bmt to CSNs. Taken together with the methylation status of other amino acid constituents of CSNs, it seems more likely that the first module might take up d*-*Ala. Consistent with our previous suggestion (1), Bmt will be the substrate recognized by the fifth module of SimA (Fig. 4B). This suggestion can be supported, at least in part, from the adenylation domain (A domain) substrate specificity and phylogenetic analyses which show that the A5 domain is highly divergent from other domains (Fig. S5A). The A2, A3, A8, and A10 domains are clustered together and have the same substrate-specific signature for recognition of leucine residues. Consistent with the divergent substrate-specific signatures, the A1 domain for d*-*Ala and the A11 domain for l*-*Ala were not clustered together (Fig. S5A).

10.1128/mBio.01211-18.5 FIG S5 Phylogenetic analysis of the adenylation and condensation domains retrieved from selected NRPSs. (A) Phylogenetic and substrate-specific signature analysis of SimA adenylation domains. Substrates: Nva, norvaline; Abu, aminobutyric acid; Sar, sarcocine. (B) Phylogenetic analysis of the condensation domains of SimA and selected NRPSs from different fungi. The NRPS BEAS from Beauveria bassiana is responsible for the biosynthesis of beauvericin. DtxS1 from *Metarhizium robertsii* is responsible for the biosynthesis of destruxins. EcdA from *Emericella rugula* is responsible for echinocandin biosynthesis, and TqaA from *Penicillium aethiopicum* is responsible for the biosynthesis of the indole alkaloid fumiquinazoline F. Download FIG S5, TIF file, 1.33 MB. Copyright © 2018 Yang et al. 2018 Yang et al. This content is distributed under the terms of the Creative Commons Attribution 4.0 International license.

Cyclization of the linear peptidyl precursors is usually mediated by a terminal condensation (C_T_) domain of fungal NRPSs (21). The carboxyl terminus of SimA also contains a condensation domain (C domain) (i.e., C12 domain). To reveal the potential cyclization function of the C12 domain, C domains of SimA and those fungal NRPSs responsible for the biosynthesis of cyclic depsipeptides were retrieved for phylogenetic analysis. The results indicated that the C12 domain of SimA is grouped within a lineage containing only the terminal C domains of different NRPSs implicated in peptidyl cyclization (Fig. S5B). Thus, the C12 domain of SimA is likely a C_T_ domain that mediates the final cyclization process to produce CsA and its analogs (Fig. 4B).

### Cyclophilin and transporter genes mediate fungal self-protection against CSNs.

CypA is the immediate receptor of CsA in humans (3). Within the biosynthetic gene cluster, *SimC* encodes a cyclophilin. Deletion of *SimC* had no obvious effect on CSN biosynthesis in *T. inflatum* (Fig. 1C). SimC is similar to human cyclophilin A (NCBI:protein accession no. P62937; 62% amino acid identity), and the gene was highly transcribed by the fungus grown in a CSN induction medium (Fig. 3A), evidence suggesting that SimC plays a role in cell self-protection from CSNs (3). To test this, we performed growth rate assays on a potato dextrose agar (PDA) with or without the addition of CsA for 2 weeks (Fig. S6A). Relative to the WT, the growth rate of the Δ*SimC* mutant was significantly reduced, whereas the growth rate of the Δ*SimA* mutant was significantly increased (*P  < *0.001) (Fig. S6B). Therefore, we suggest that as CSNs are toxic to other fungi and also to the producing fungus *T. inflatum*, SimC may function to bind CSN to reduce toxicity and increase cell tolerance of the accumulated cyclopeptides. The results also indicated that the addition of CsA significantly (*P < *0.001) inhibited the growth of the examined WT, Δ*SimC*, Δ*SimA*, and Δ*SimD* strains compared with the growth on PDA. Besides SimC, nine additional cyclophilin genes, including a highly similar paralog TINF04375 (63% identity), were identified in the genome of *T. inflatum* (1). These proteins are separated in different lineages (Fig. S7). It is not clear whether the closely related cyclophilins may play an additional or redundant role in fungal self-protection.

10.1128/mBio.01211-18.6 FIG S6 Self-inhibition and transportation assay of cyclosporin production in *T. inflatum*. (A) Phenotyping of fungal growth on PDA with or without the addition of CsA. Spore suspensions (1 × 10^7^ spores/ml) were individually inoculated (2 μl each) on PDA amended with or without CsA (at a final concentration of 150 μg/ml) and incubated for 14 days. (B) Comparison of colony diameters between WT and mutants after the growth shown in panel A. Values are means plus SE. Values that are significantly different are indicated by asterisks as follows: **, *P < *0.01; *, *P < *0.05. (C) Comparison of cellular accumulation of CsA between WT and Δ*SimD*. DW, mycelium dry weight. (D) Comparison of extracellular accumulation of CsA between WT and Δ*SimD* strains. Values are means plus SE. Values that are significantly different (*P  < *0.05) are indicated by an asterisk. Download FIG S6, TIF file, 2.10 MB. Copyright © 2018 Yang et al. 2018 Yang et al. This content is distributed under the terms of the Creative Commons Attribution 4.0 International license.

10.1128/mBio.01211-18.7 FIG S7 Phylogenetic analysis of the cyclophilin proteins encoded by *T. inflatum* genes (shown in boldface type) and selected insect-pathogenic fungi. The cyclophilin genes characterized in *Beauveria bassiana* are also shown in boldface type. The sources of the proteins are indicated by prefixes as follows: CCM, *Cordyceps militaris*; OCS, *Ophiocordyceps sinensis*; MAA, *Metarhizium robertsii*; CCAD, *Cordyceps cicadae*; BBA or Bb, *Beauveria bassiana.* Download FIG S7, TIF file, 0.49 MB. Copyright © 2018 Yang et al. 2018 Yang et al. This content is distributed under the terms of the Creative Commons Attribution 4.0 International license.

We found that deletion of the ABC transporter gene *SimD* resulted in a higher (*P = *0.0147) level of cellular CsA accumulation but a lower (*P = *0.0287) level of CsA in the culture filtrates compared with the WT (Fig. S6C and D). The growth rates of WT and Δ*SimD* mutant had no obvious difference on PDA, but the latter was significantly (*P = *0.0023) inhibited on CsA-amended PDA compared with the WT (Fig. S6B). The results indicated that SimD functions as an exporter and may jointly contribute to the resistance of CSNs in *T. inflatum*.

### CsA production benefits fungal competition against other fungi.

**FIG 5:**
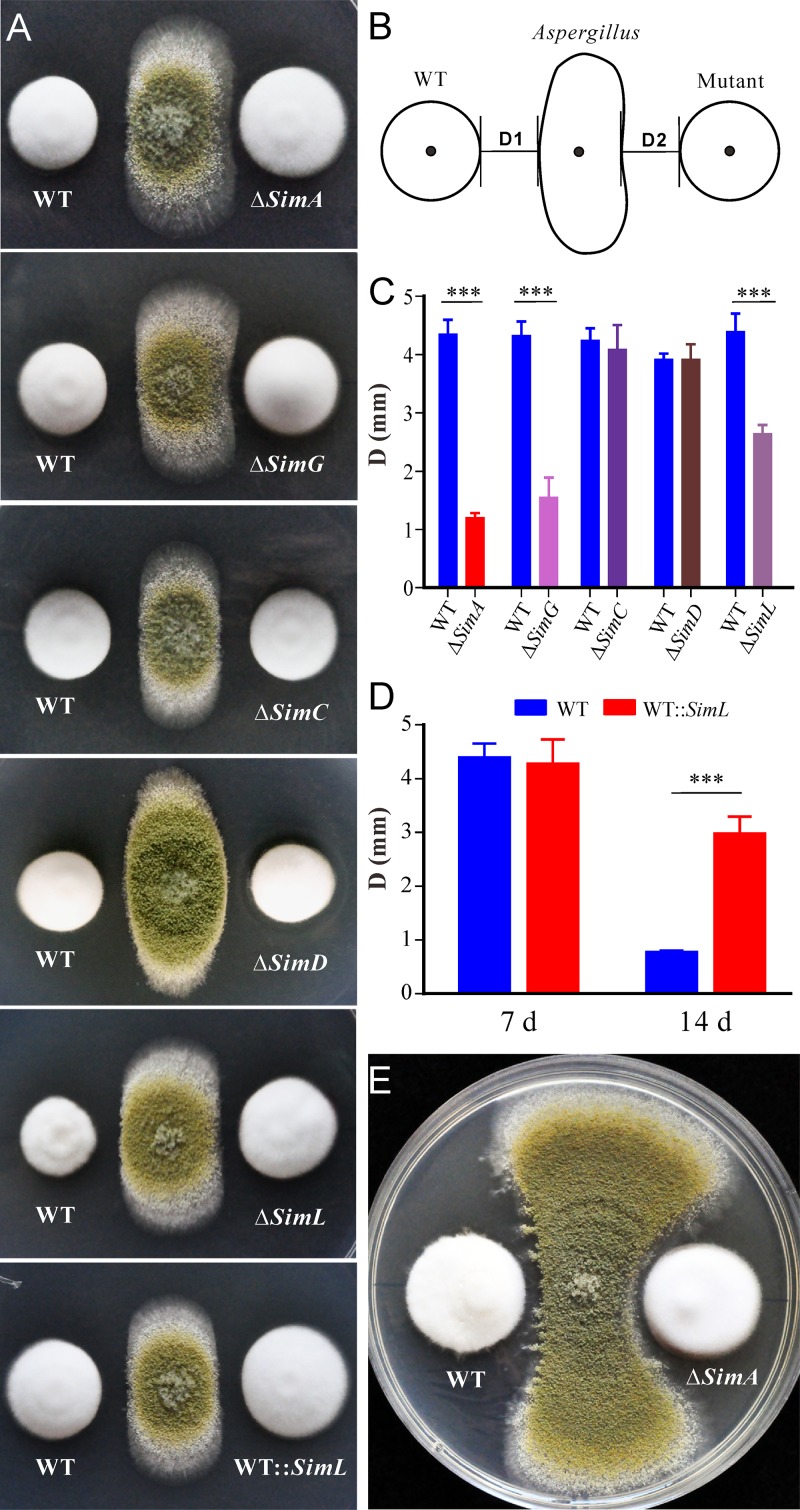
Antifungal effect of CsA production. (A) Fungal cocultivation tests. The WT and mutants of *T. inflatum* were inoculated on PDA plates in parallel for 3 days, and the strain of A. flavus was then inoculated between the two *T. inflatum* colonies for 4 days. (B) Schematic diagram showing how the colony edge distances between the WT *T. inflatum* and *Aspergillus* (D1) or between the *T. inflatum* mutant and *Aspergillus* (D2) were measured. (C) Comparison of the colony edge distances between strains. Values are means plus SE. Values that are significantly different (*P < *0.001 by two-tailed *t* test) are indicated by a bar and three asterisks. (D) Comparison of the colony edge distances between WT and WT::*SimL* strains after different incubation times (7 or 14 days [d]). Values are means plus SE. ***, *P < *0.001. (E) Representative phenotypes of a fungal pair after inoculation and 3 weeks of growth of *T. inflatum*.

Given that CsA has antifungal activity (3), we performed fungal competition tests by cocultivation of the WT and different mutants of *T. inflatum* with the saprophytic fungus Aspergillus flavus (Fig. 5A). After measuring the colony edge distance 1 week postinoculation (Fig. 5B), comparative analysis revealed that the distance between the WT *T. inflatum* and *Aspergillus* was significantly larger (*P < *0.001) than those between CsA-nonproducing mutants (Δ*SimA*, Δ*SimG*, and Δ*SimL*) and the mold fungus (Fig. 5C). The pairwise comparison between WT and CsA-producing mutants (*SimC*, *SimD*, and WT::*SimL* ) found no difference 1 week postinoculation (Fig. 5C and D). However, the overexpression mutant of *SimL* inhibited *Aspergillus* growth more significantly (*P < *0.001) than did the WT after growth for 14 days (Fig. 5D). A competitive advantage could be more clearly observed such as between WT and Δ*SimA* after the fungi were grown for up to 3 weeks (Fig. 5E). Further cocultivation tests against other insect-pathogenic fungi, i.e., the close relatives/competitors of *Tolypocladium*, also revealed that the WT demonstrated stronger antifungal activity than the Δ*SimA* mutant did (Fig. S8). Thus, the production of CSNs can benefit *T. inflatum* by conferring its ability to outcompete other fungi in the environment.

10.1128/mBio.01211-18.8 FIG S8 Cocultivation test of *T. inflatum* with other insect-pathogenic fungi. The strains of other fungi were inoculated on PDA plates in the middle for 2 days, and the WT and Δ*SimA* strains of *T. inflatum* were then inoculated in parallel for 7 days. MRO, *Metarhizium robertsii*; CCA, *Cordyceps cicadae*; CMI, *C. militaris*; BBR, *Beauveria brongniartii*. Download FIG S8, TIF file, 1.47 MB. Copyright © 2018 Yang et al. 2018 Yang et al. This content is distributed under the terms of the Creative Commons Attribution 4.0 International license.

### CsA production is required for fungal full virulence.

**FIG 6:**
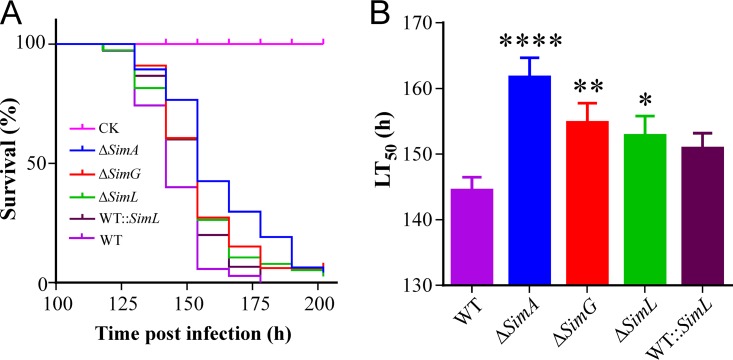
Insect bioassays. (A) Survival of insects after injection with the spores of the WT and different mutants. Control insects (CK) were injected with 0.05% Tween 20. (B) Comparison of the LT_50_ values for the WT and different mutants. Values are means plus SE. Values that are significantly different from the value for the WT by log rank tests are indicated by asterisks as follows: ****, *P < *0.0001; **, *P = *0.0087; *, *P = *0.0182.

To determine the contribution of CsA production to fungal virulence, we conducted insect bioassays by injecting a spore suspension into the last instar larvae of wax moth (Galleria mellonella) (Fig. 6A). The estimation of median lethal time (LT_50_) indicated that the LT_50_ values of Δ*SimA*, Δ*SimA*, and Δ*SimL* mutants were significantly higher than that of the WT, i.e., the null mutants became less virulent. However, the difference between WT and WT::*SimL* was insignificant (Fig. 6B). Consistent with the suppressive effect of CsA on insect immunities (16), the data indicated therefore that CsA production plays a role in fungal infection of insect hosts.

## DISCUSSION

After the development of CsA as a commercial immunosuppressant drug, decades-long efforts have attempted but failed to uncover the full mechanism of CsA biosynthesis (5). In this study, we propose the biosynthetic mechanism, regulation control, self-protection strategy, and chemical ecology of CSN production in *T. inflatum*. Ten genes were selected for loss-of-function studies by excluding a misannotated artifact and four hypothetical genes. We verified that the bZIP-type transcription factor (TF) SimL, but not the zinc finger TF TINF00183, mediates the pathway-specific regulation of cyclosporine (CSN) production in *T. inflatum*. Thus, overexpression of *SimL* could substantially increase the biosynthetic titer of CSNs in the fungus. Taken together with the RT-PCR analysis of gene expression levels in Δ*SimL* and WT::*SimL* strains (Fig. 3A), the genes located upstream of *SimA* and downstream of *SimL* are unlikely to belong to the CsA biosynthetic cluster.

We found that deletion of the thioesterase (TE)-like gene *SimE* had no obvious effect on CSN production in *T. inflatum* (Fig. 1C). In bacteria, however, deletion of the free TE gene within a cluster significantly reduced metabolite production (22). It has been found that additional TE genes in either the PKS or NRPS gene cluster could not compensate for the catalysis of compound termination/cyclization mediated by the integral TE domain found at the end of core PKS or NRPS enzymes. Instead, the free TE gene may fulfill an editing function during metabolite biosynthesis (22). We found that the terminal C domain of SimA could be grouped together with those C_T_ domains being functionally verified to catalyze the cyclization of macrocycles, e.g., the C_T_ domain of the NRPS TqaA from *Penicillium aethiopicum* (21, 23). It is suggested therefore that the terminal C domain of SimA might contribute to the release and cyclization of CSNs. The exact function of *SimE* remains to be determined. In addition, since the expression of the hypothetical genes *SimF* and *SimH* is similarly regulated by SimL, future studies are still required to determine the functions of these predicted protein genes. Considering that the cluster genes are differentially regulated by SimL in the WT strain and that the expression of *SimC* and *SimK* could still be detected in the Δ*SimL* mutant (Fig. 3A), the possibility that additional TF(s) may be involved in jointly controlling the biosynthesis of CSNs cannot be ruled out.

Nine of eleven amino acids in CsA are nonproteinogenic, including the d*-*Ala, Bmt, l-aminobutyric acid (Abu), sarcocine (Sar) (*N*-methylglycine), and *N-*methylated leucine and methylvaline. *In situ N*-methylation can be mediated by the NM domains encoded in the corresponding modules of SimA (Fig. 4B). Our genome survey indicated that a single copy of the AlaR gene (*SimB*) is present in the genome of *T. inflatum*. Thus, consistent with the biochemical study using the purified AlaR enzyme (6), gene deletion and substrate feeding assays confirmed that SimB contributes to d*-*Ala conversion (Fig. 2A). Intriguingly, we found that trace amounts of CsA could still be produced by the *ΔSimB* mutant and the double mutant with both the *SimB* and threonine aldolase (*TA)* genes deleted. In contrast, deletion of *ToxG* (42% identity to SimB at the amino acid level) in the plant pathogen *Cochliobolus carbonum* completely abolished the production of the d*-*Ala-containing form of HC toxin (24). No alternative substitute is found in place of d*-*Ala in the structures of CSNs (3). Thus, an additional source of d*-*Ala remains to be determined in *T. inflatum*. It is also unclear why feeding d*-*Ala failed to restore the abilities of the *ΔSimB* and *ΔSimBΔ06009* mutants to produce the WT level of CsA (Fig. 2B). Structurally, CsA (Abu) differs from CsB (Ala) and CsC (Thr) only at position 6 (Fig. 4B). Abu can be converted from Thr through the function of threonine deaminase (25). A yeast ILV1-like threonine deaminase is present in *T. inflatum* (TINF04164, 50% amino acid identity). This gene is not present in the *SimA* cluster, implying that Abu could be synthesized outside the cluster for CsA biosynthesis. Regarding the PKS pathway for Bmt biosynthesis, different DAD-chromatographic profiles (isoabsorbance plots) could at least be observed between the Δ*SimG* mutant and the Δ*SimI/*Δ*SimJ* mutant and between the Δ*SimI* and Δ*SimJ* mutants (see Fig. S4 in the supplemental material). Future efforts are still required to determine the oxidation function of SimI and verify the structure of the proposed intermediates.

Accumulated evidence has indicated that the small molecules produced by different fungi as well as bacteria play important roles in the environmental adaptation and competitive advantage of the organisms producing the molecules. However, except for the perceived biological properties, the ecological importance of many metabolites is largely unknown (26). A few secondary metabolites produced by insect-pathogenic fungi have been established to contribute to fungal virulence against insect hosts such as the destruxins produced by *Metarhizium* species (27) and the beauvericin and oosporein biosynthesized by Beauveria bassiana (28, 29). The oosporein produced by *Beauveria* could also help the fungus to outcompete bacterial growth during fungal colonization of insect hosts (30, 31). Consistent with the immune inhibition and insecticidal effect of CsA on insects (16, 32), our insect bioassays revealed that abolishment of CSN production impaired fungal virulence against insect hosts. In this study, we also established that the production of CSNs can enable the CSN-producing fungus *T. inflatum* to outcompete other fungi in the environment. However, the exact mechanisms of CSN ecological functions required further investigations.

In conclusion, we report the biosynthesis of CSNs that is regulated by a bZIP-type TF SimL in *T. inflatum*. It is indicated in this study that the cyclophilin and transporter genes encoded in the *SimA* cluster contribute to the self-protection/tolerance of CSNs in the CSN-producing fungus. On the other hand, CSN production benefits the fungus by allowing it to outcompete other fungi and facilitate fungal infection of insect hosts. In addition to suggesting the CSN biosynthetic pathway, the results of this study advance our understanding of the ecological role of this important drug molecule to fungal adaptations in the environment.

## MATERIALS AND METHODS

### Fungal strains and reagents.

The wild-type (WT) strain NRRL 8044 (ATCC 34921) of Tolypocladium inflatum (1) was used to generate the gene deletion mutants. Both the WT and mutants were maintained either on potato dextrose agar (PDA) (BD Difco) or in a Sabouraud dextrose broth (SDB) (BD Difco). For induction of CsA production, the WT or mutants was induced in the cyclosporine (CSN) induction medium containing fructose (fructose CSN induction medium) [fructose, 30 g/liter; (NH_4_)_2_HPO_4_, 6 g/liter; yeast extract, 5 g/liter; CaCl_2_·2H_2_O, 1.32 g/liter; MgSO_4_·7H_2_O, 2.05 g/liter; FeSO_4_·7H_2_O, 27.4 mg/liter; ZnSO_4_·7H_2_O, 17.8 mg/liter; CoCl_2_·6H_2_O, 27.5 mg/liter; CuSO_4_·5H_2_O, 3.1 mg/liter] adjusted from a previous study (17). The strains of Aspergillus flavus (NRRL 3357), Metarhizium robertsii (ARSEF 23) (33), Cordyceps cicadae (CCAD02) (34), Cordyceps militaris (Cm01) (35), and Beauveria brongniartii (RCEF 3172) (36) were used for antifungal assays between WT and null mutants of *T. inflatum*. The standards of cyclosporin A (CsA) and d-Ala were ordered from Sigma-Aldrich (USA), and CsB and CsC were purchased from Santa Cruz Biotechnology (USA).

### Gene deletion, overexpression, and fungal transformation.

To determine the functions of the clustered genes, gene deletions were individually performed by using *Agrobacterium*-mediated transformation of *T. inflatum* (37). To generate the deletion vectors, the 5′- and 3′-flanking regions of the target gene were ampliﬁed by PCR using different primer pairs (see Table S2 in the supplemental material). For example, primers SimA*-*U1/SimA-U2 (U stands for upper strand) were used to amplify the *SimA* upstream region, and primers SimA*-*L1/SimA-L2 (L stands for lower strand) were used to amplify the *SimA* downstream region. The PCR fragments obtained were treated with the restriction enzymes, purified, and then cloned into the same enzyme-treated binary vector pDHt-SK-Bar (28) for *Agrobacterium-*mediated transformation of the WT strain. For double deletion of *SimB* and the putative threonine aldolase (TA) gene *TINF06009*, the flanking regions of *TINF06009* were amplified by fusion PCRs with the ClonExpress II one step cloning kit (Vazyme, China), and the products were cloned into the binary vector pDHt-SK-Ben (28) for transformation of the *ΔSimB* mutant. The drug resistance colonies were verified by PCR, and reverse transcription-PCR (RT-PCR) was performed to verify gene deletions after single spore isolation. For overexpression of the transcription factor SimL in the WT strain, the full-length open reading frame (ORF) of this gene was amplified using the genomic DNA of *T. inflatum* as a template by fusion PCRs with different primers using the ClonExpress kit. The gene was made under the control of the constitutive GpdA gene (*TINF02918*) promoter, and the cassette was cloned into the pDHt-SK-Bar plasmid for transformation of the WT strain of *T. inflatum*.

10.1128/mBio.01211-18.10 TABLE S2 Primers designed and used in this study. Download Table S2, PDF file, 0.06 MB. Copyright © 2018 Yang et al. 2018 Yang et al. This content is distributed under the terms of the Creative Commons Attribution 4.0 International license.

### Gene expression profiling and scanning of the putative binding site of SimL.

To determine gene expression control by SimL, the WT, Δ*SimL*, and WT::*SimL* strains were grown in the fructose CSN induction medium for 10 days. The mycelia were harvested, washed twice with sterile water, and homogenized in liquid nitrogen for RNA extraction with the TransZol UP Plus RNA kit (Transgen Biotech, China) by following the manufacturer’s protocol. The RNA (20 μg each) was then converted to cDNA using the ReverTra Ace quantitative PCR (qPCR) real-time (RT) master mix (Toyobo Life Science, Japan). Semiquantitative RT-PCR analysis was performed using the primer pairs for different genes (Table S2). A β-tubulin gene (*TINF09088*) of *T. inflatum* was amplified as a reference. The conserved binding motif of the fungal basic leucine zipper (bZIP)-type transcription factor (TF) was identified to be 5′-TGACG-3′ (20). To examine the presence or absence of this motif, the promoter region (1 to 1.5 kb upstream of the start codon) of the cluster gene was scanned. Each identified motif was extracted together with its flanking sequences (five nucleotides) to generate the sequence logo using WebLogo 3 (38).

### Induction of CSN production and chromatography analysis.

The conidial spores of the WT and different mutants of *T. inflatum* were harvested from *T. inflatum* cultures grown on PDA plates for 4 weeks and suspended in 0.05% Tween 20 to a final concentration of 1 × 10^8^ spores/ml. The spores were inoculated (50 μl each) into the fructose CSN induction medium (50 ml in each flask; pH 5 to 6) and incubated in a rotatory shaker at 25°C and 200 rpm for 10 days. For d*-*Ala feeding assays, the cultures were also grown in the fructose CSN induction medium supplemented with d-Ala at a final concentration of 20 mM for 10 days. There were three replicates for each strain. The mycelia of each sample were harvested under vacuum, freeze-dried, and homogenized into fine powders. An equal amount of sample (20 mg each) was individually extracted with 500 μl of methanol at 4°C for 2 days assisted with sonication treatments. The samples were then centrifuged at a maximum speed for 10 min, and the supernatants were collected for high-performance liquid chromatography (HPLC) analysis at 210 nm using a LC-20AD system (Shimadzu, Japan) equipped with an SPD-20A UV-visible (UV-Vis) detector and a C_18_ reverse-phase column (particle size, 5 μm; length, 4.6 by 250 mm; Agilent Eclipse XDB, USA). Aliquots (10-μl aliquots) of samples were eluted with the deionized water (solution A) and acetonitrile (solution B, 65 to 100% acetonitrile) at a flow rate of 0.8 ml/min. Quantification of CsA production was performed by calibration to the standard curve generated using the CsA standard.

### Bmt purification and substrate feeding.

The intermediates and (4*R*)-4-[(E)-2-butenyl]-4-methyl-l-threonine (Bmt) produced by the polyketide synthase (PKS) pathway have no UV observance signals. To determine the compound structures, the Δ*SimA* mutant (having the highest accumulation level of Bmt) was grown in the fructose CSN induction medium in 1-liter flasks on a large scale (5 liters in total). The cultures were incubated at 25°C and 200 rpm for 2 weeks. The culture filtrates were collected, concentrated, and then extracted with methanol three times. Mycelial samples were freeze-dried and extracted with methanol also. The samples were first eluted with the preparative HPLC (LC-6AD; Shimadzu, Japan) system equipped with an Inestsil ODS C_18_ column (particle size, 5 μm; length, 10 by 250 mm; GL Sciences, Japan) and a FRC-10A fraction collector. The samples were eluted with deionized water (solution A) and acetonitrile (solution B, 5 to 100% acetonitrile) at a flow rate of 3 ml/min. The eluents were collected every 5 min, and the fractions were then subjected to purification using the Waters Acquity ultraperformance liquid chromatographic (UPLC) system equipped with an Acquity QDa MS (mass spectral) detector (Waters) and an Xbridge Prep C_18_ column (particle size, 5 μm; length, 19 by 150 mm; Waters, USA). The samples were eluted with 0.1% formic acid and acetonitrile (0 to 10 min, 2 to 8%; 10 to 15 min, 8 to 20%; 15 to 16 min, 20 to 95%) at a flow rate of 15 ml/min. The fractions containing the compound with the molecular weight of Bmt, and compounds b1 to b3 were collected. These samples were further purified with the system and eluted with 0.1% formic acid and acetonitrile (0 to 6.5 min, 10 to 12%; 6.5 to 12 min, 90%) at a flow rate of 10.8 ml/min. The purified samples were examined with an Agilent liquid chromatography (LC)-mass spectrometry (MS) system (6210 time of flight [TOF]/quadrupole time of flight [Q-TOF] LC-mass spectrometer; Agilent, USA) for purity analysis. After different trials, only Bmt was successfully collected. The compound (4 mg) was dissolved in D_2_O for one-dimensional (1D) and 2D nuclear magnetic resonance (NMR) structure analysis with the Bruker Advanced III-500MHz system. After structure analysis, Bmt was used for feeding assays. Thus, the spores of Δ*SimG*, Δ*SimI*, and Δ*SimJ* mutants were inoculated in the fructose CSN induction medium (15 ml each in 100-ml flasks) supplemented with Bmt at a final concentration of 85 μM. There were three replicates for each mutant, and the WT strain was inoculated in parallel for comparative analysis. After incubation for 2 weeks, the mycelia were harvested and extracted with methanol for HPLC analysis of CsA production.

### Phylogenetic analysis.

To determine the substrate specificity of the adenylation domains (A domains) and the potential cyclization role of the terminal condensation domain (C_T_ domain) of SimA, both the A and condensation (C) domains were retrieved based on the analysis with the program antiSMASH (ver. 3.0) (39) for phylogenetic analysis. Substrate-specific signatures of SimA A domains were predicted with the program NRPSpredictor2 (40). Additional C domains were also retrieved from the nonribosomal peptide synthetase (NRPS) DtxS1 for destruxin biosynthesis in *Metarhizium* spp. (27), and the beauvericin nonribosomal cyclodepsipeptide synthetase (BEAS) for the production of beauvericin in *B. bassiana* (29) as well as the cyclization C_T_ domains of *Penicillium aethiopicum* TqaA (21, 23) and *Emericella rugula* EcdA (41). The A- or C-domain sequences were aligned with the program Clustal X (ver. 2.1) (42), and neighbor-joining (NJ) trees were generated with the program MEGA 7.0 (43) using a Dayhoff model, pairwise deletion for gaps/missing data, and 1,000 bootstrap replications to test the phylogeny. Cyclophilin can bind CsA for cell protection/tolerance (3). Together with SimC, there are 10 cyclophilin genes encoded in the genome of *T. inflatum* (1). Different cyclophilins were selected from the close relatives of *T. inflatum* for phylogenetic analysis, including those from the insect pathogens *Beauveria bassiana* (44), *Metahizium robertsii* (33), *Cordyceps militaris* (35), *Cordyceps cicadae* (34), and *Ophiocordyceps sinensis* (45). A NJ tree was generated using the same parameters indicated above.

### Antifungal activity assay.

CsA can bind cyclophilin to mediate antifungal activity (3). To test the effect of cyclophilin SimC and transporter SimD on cell protection, the WT, Δ*SimA*, Δ*SimC*, and Δ*SimD* strains of *T. inflatum* were grown on PDA amended with CsA or without CsA (supplemented at a saturated level in the medium). The colony sizes were then measured 12 days after inoculation, and the differences were compared for the WT and individual mutant strains to determine the effect of CsA on fungal growth. To determine the effect of CSN production on fungal competition with other fungi, both the WT and mutants of *T. inflatum* were inoculated on PDA plates (9 cm in diameter) in parallel for 3 days, and A. flavus spores (2 μl of a suspension containing 1 × 10^7^ spores/ml) were then inoculated in the middle of two colonies for pairing and competitive growth. Colony edge distances were measured to determine the differences between WT and *Aspergillus* and between mutants and *Aspergillus* after inoculation of *T. inflatum* for 1 or 3 weeks. Similar experiments were done by competition tests with other insect-pathogenic fungi, including *M. robertsii*, *C. militaris*, *C. cicadae.* and *B. brongniartii*. Each experiment has three replicates, and the difference between samples was examined using a Student’s *t* test.

### Insect bioassays.

Insecticidal activity of CSNs was first demonstrated in mosquito larvae (32). To further determine the metabolite contribution to fungal virulence, insect bioassays were conducted by injection of the last instar larvae of wax moth. The spore suspensions of the WT, Δ*SimA*, Δ*SimG*, Δ*SimL*, and WT*::SimL* strains were prepared from the cultures grown on PDA plates for 4 weeks. The spore suspensions of the strains were placed in 0.05% Tween 20 to a final concentration of 2 × 10^6^ spores/ml. Insect larvae were injected individually in the second proleg with the spore suspension (10 μl each). There were three replicates (15 insects per replicate) for each strain, and the experiments were repeated twice. The control insects were injected with 0.05% Tween 20. Mortality over time was recorded for each strain, and the median lethal time (LT_50_) values were estimated and statistically compared for the WT and each mutant by Kaplan-Meier analysis with SPSS (ver. 22.0) (46).
