# Supplementary figures and images for: Cyclosporine Biosynthesis in Tolypocladium inflatum Benefits Fungal Adaptation to the Environment
Source: mBio. 2018 Oct 2;9(5):e01211-18. doi: 10.1128/mBio.01211-18 (PMC6168864; doi:10.1128/mBio.01211-18)

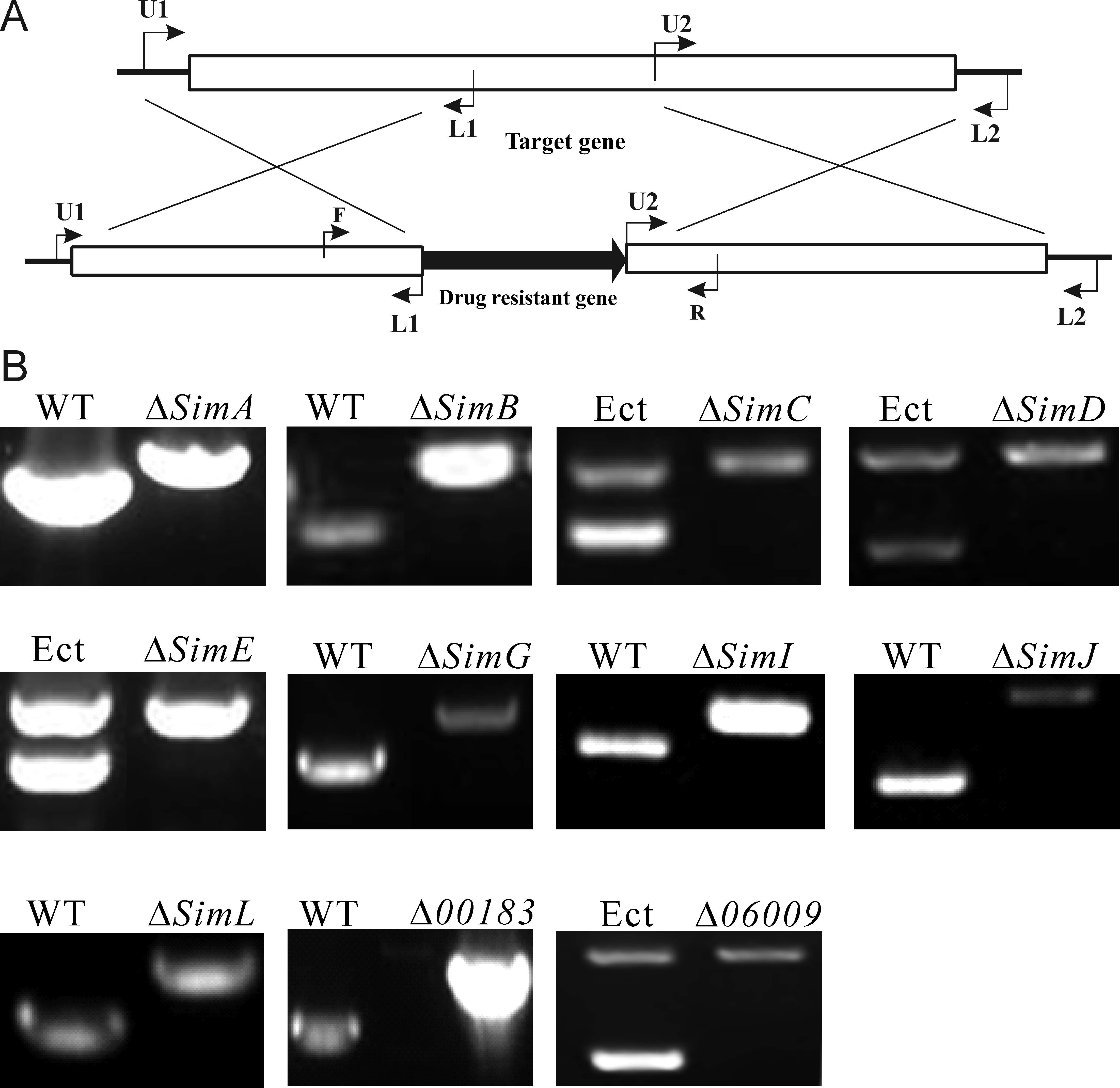

Supplement: FIG S1 [file mbo005184087sf1.tif]

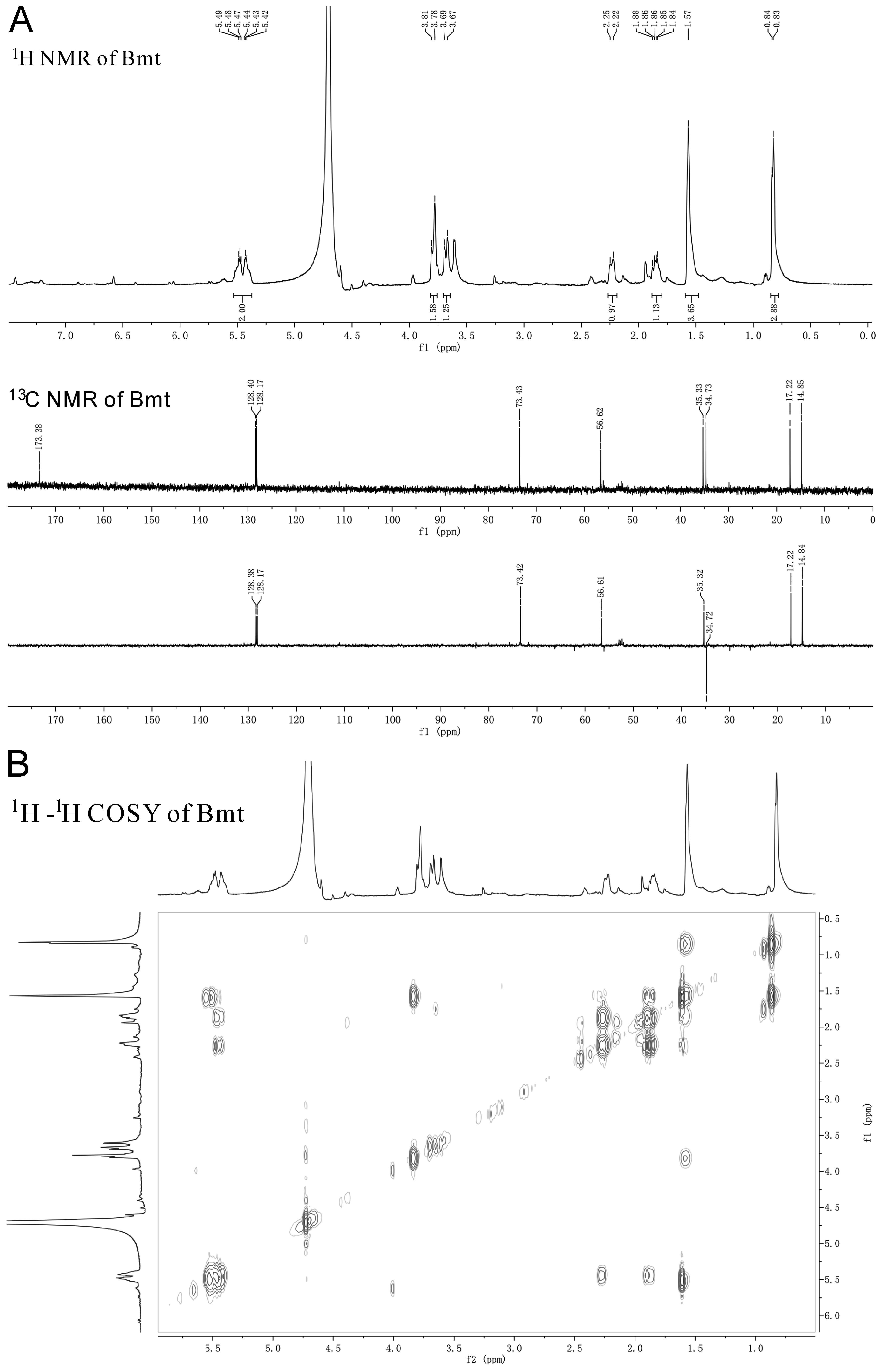

Supplement: FIG S2 [file mbo005184087sf2.tif]

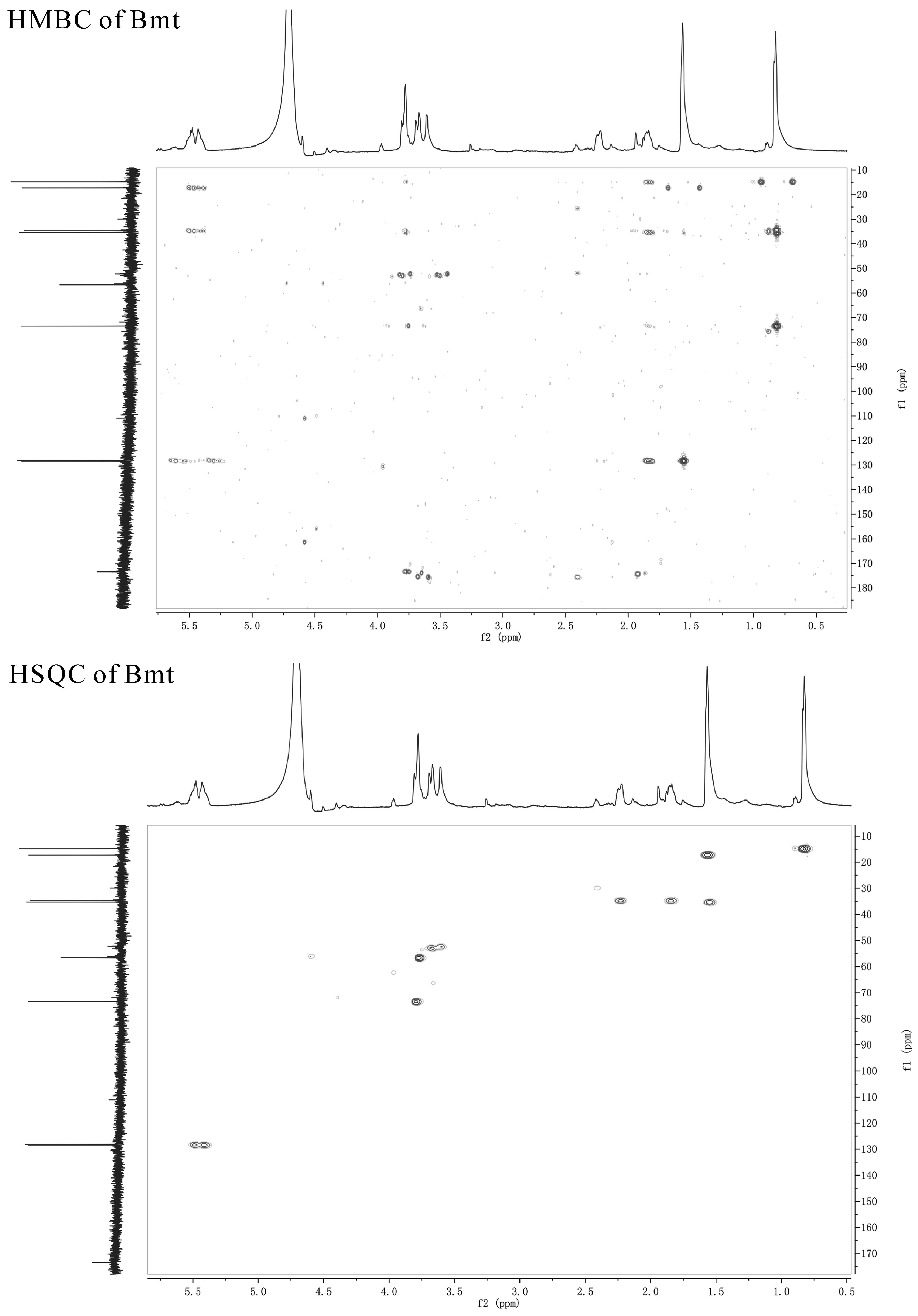

Supplement: FIG S3 [file mbo005184087sf3.tif]

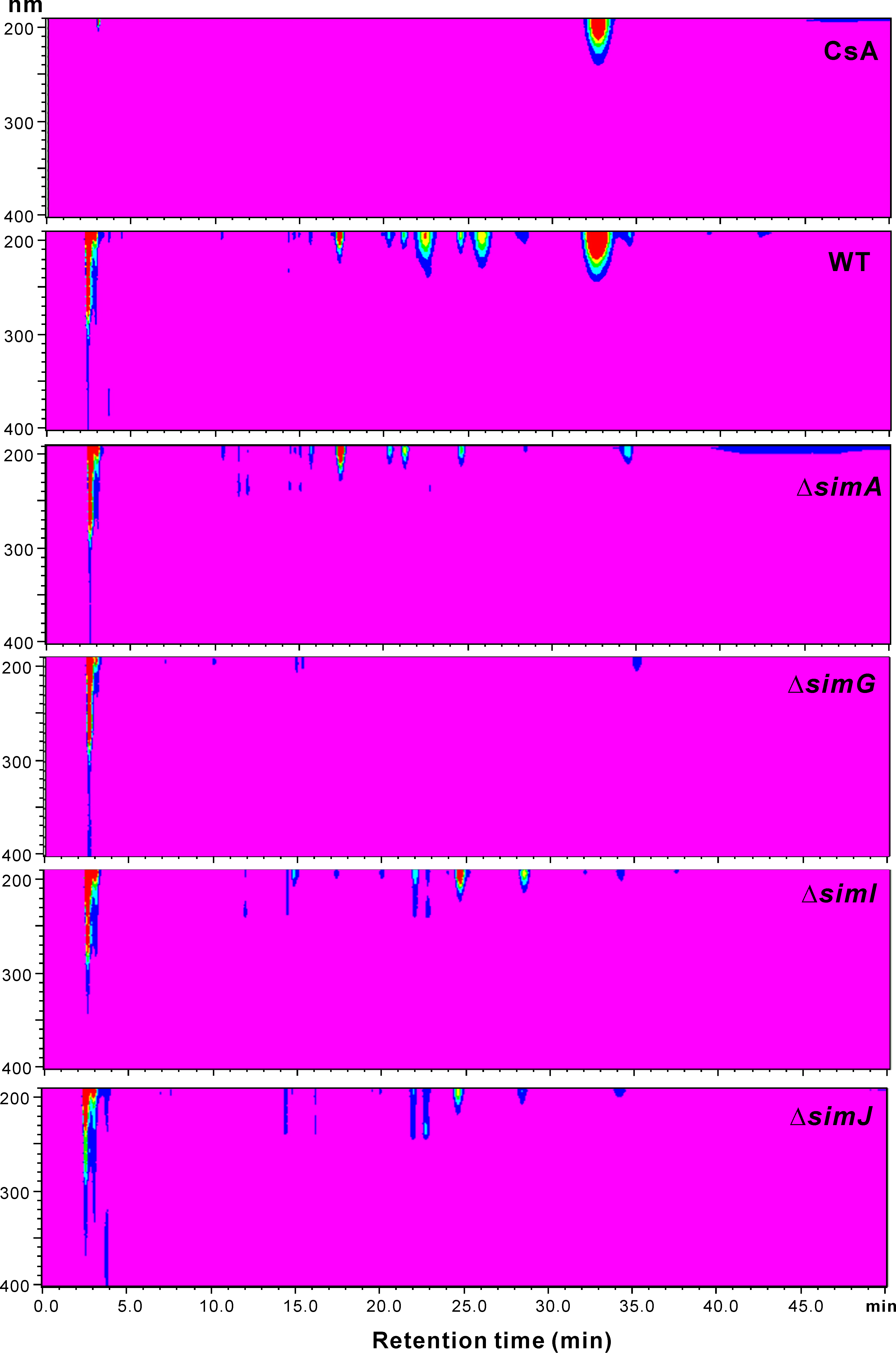

Supplement: FIG S4 [file mbo005184087sf4.tif]

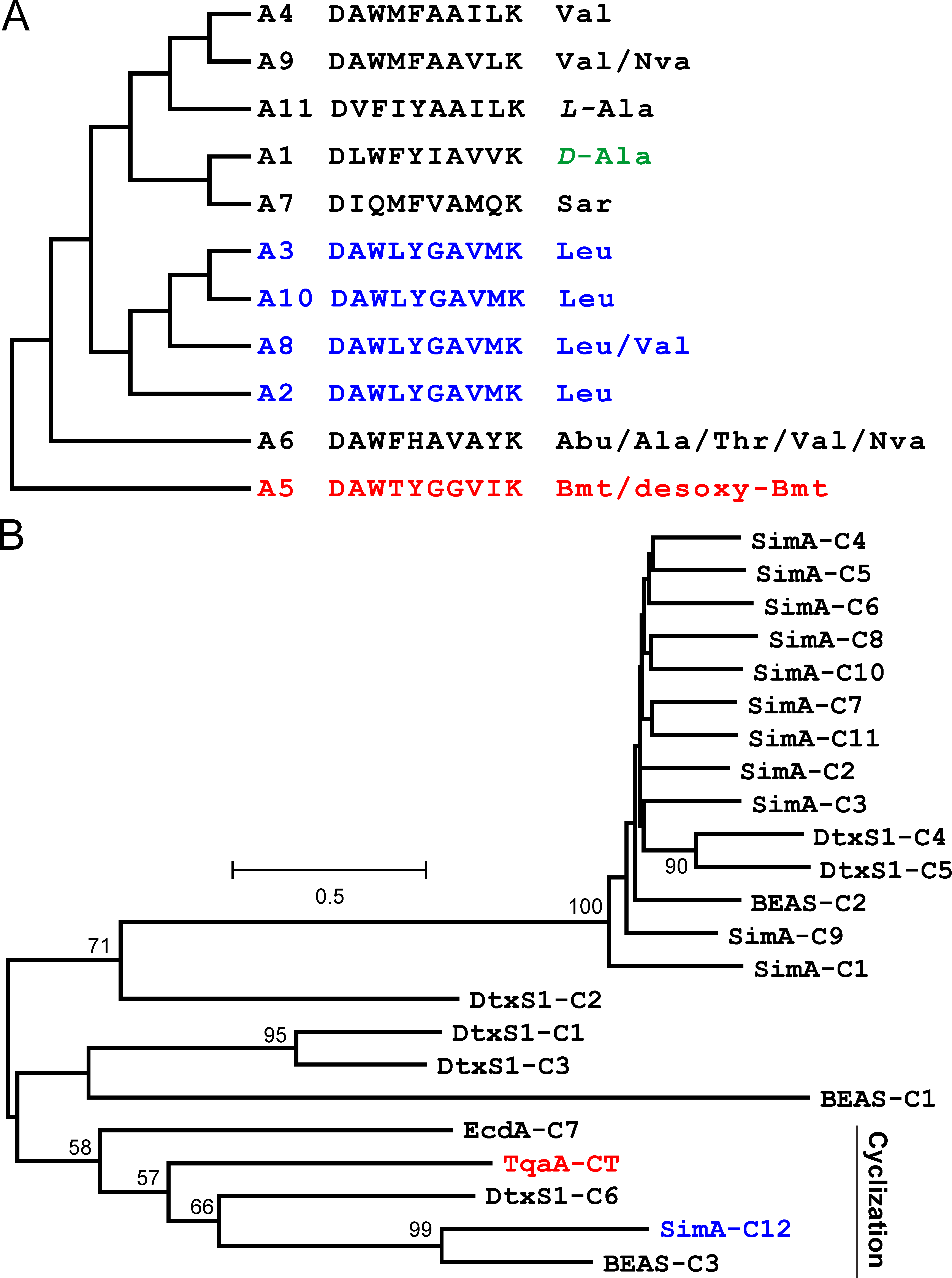

Supplement: FIG S5 [file mbo005184087sf5.tif]

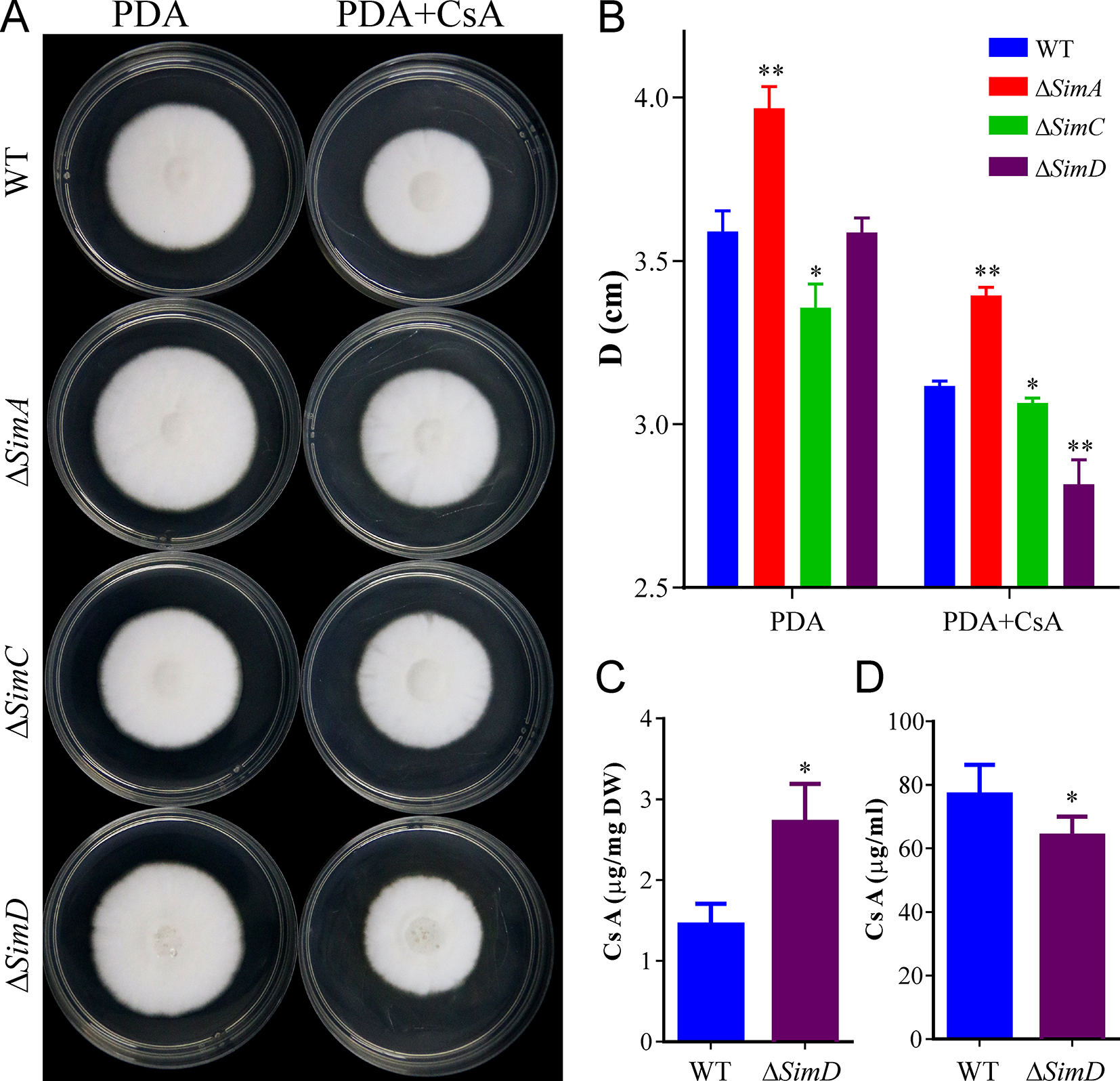

Supplement: FIG S6 [file mbo005184087sf6.tif]

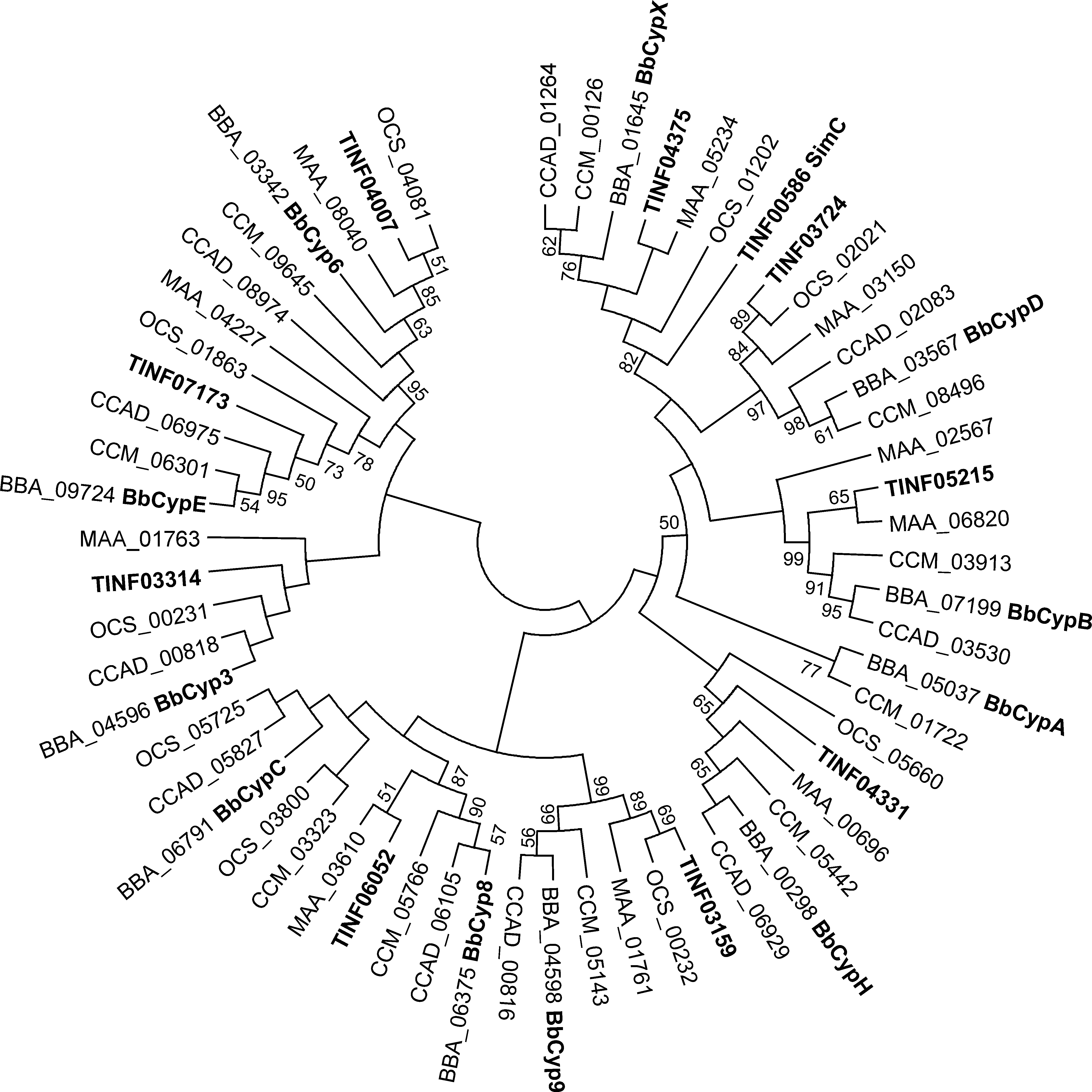

Supplement: FIG S7 [file mbo005184087sf7.tif]

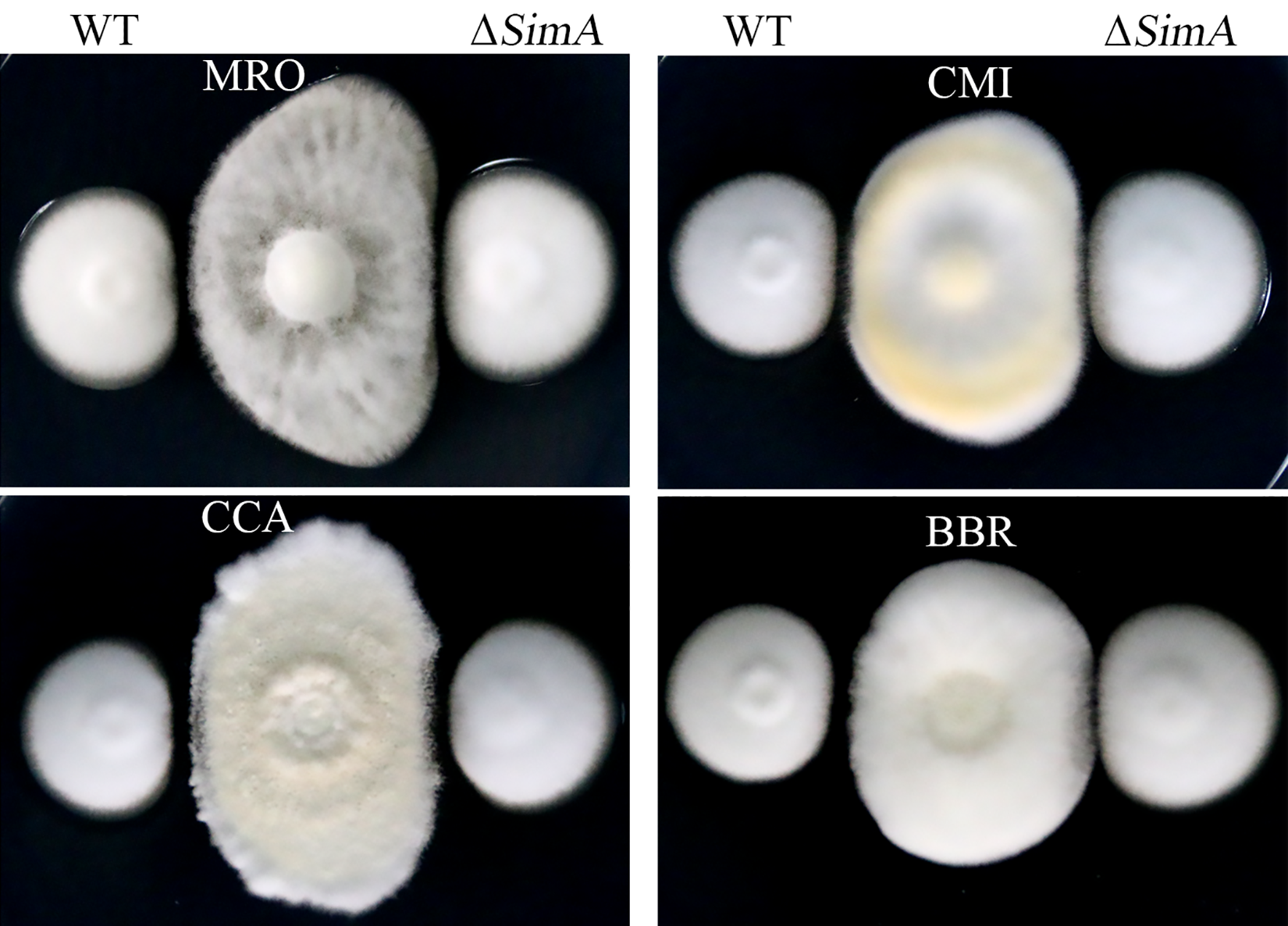

Supplement: FIG S8 [file mbo005184087sf8.tif]
