# Supplementary material for: Cyclosporine Biosynthesis in Tolypocladium inflatum Benefits Fungal Adaptation to the Environment
Source: mBio. 2018 Oct 2;9(5):e01211-18. doi: 10.1128/mBio.01211-18 (PMC6168864; doi:10.1128/mBio.01211-18)
Supplement: TABLE S1 [file mbo005184087st1.pdf]

Table S1. NMR data for identification of Bmt.[a]

No.	δ_H (mult., J in Hz)	δ_C	HMBC (H→C)	^1H-^1H COSY
**1**		173.4		
**2**	3.77[b]	56.6	1. 3, 4	
**3**	3.80[b]	73.4	1, 4, 4-CH_3, 5	4
**4**	1.55	35.3		3, 5
**4-CH_3**	0.83, d (6.0)	14.9		4
**5**	2.23, m<br>1.85, m	34.7	 4, 4-CH_3, 6, 7	6<br>4, 6
**6**	5.46, m	128.2[c]	5, 8	5
**7**	5.46, m	128.4[c]	5, 8	8
**8**	1.57, d (6.0)	17.2	6, 7	7

[a] Recorded in D_2O at 500 MHz.

[b] Signals overlapped with each other.

[c] Values with identical superscripts in each column may be interchanged.
