# Supplementary material for: Cyclosporine Biosynthesis in Tolypocladium inflatum Benefits Fungal Adaptation to the Environment
Source: mBio. 2018 Oct 2;9(5):e01211-18. doi: 10.1128/mBio.01211-18 (PMC6168864; doi:10.1128/mBio.01211-18)
Supplement: TABLE S2 [file mbo005184087st2.pdf]

Table S2. Primers designed and used in this study.

Genes	Primers	Primer sequence*	Restriction enzymes	Note
SimN TINF00183	U1	GGAATTCCCGAGCAGTGGAACTTCTTC	*EcoR* I	Gene deletion
	U2	CGGGATCCATATGTTCCGTCGCAAGAGC	*BamH* I	
	L1	GACTAGTACCGGGTAGAGGCAGAAGAC	*Spe* I	
	L2	CGAGCTCTGAACGTTGGTCAGTTGAGG	*Sac* I	
	F	GAGCCAGTCAGTCTTGGTGTC		PCR verification
	R	CGGAGAGGTGATGGATGG		
SimA TINF00159	U1	GGAATTCTATCGGGCAAATCTTCATGG	*EcoR* I	Gene deletion
	U2	TCCCCCGGGCGGTATTGAATGGGAATTGG	*Sma* I	
	L1	GCTCTAGATACCCGATGTTGAGTTGCTG	*Xba* I	
	L2	CGAGCTCCAGTTGCTACGGATGCTTGA	*Sac* I	
	F	CTGGCAGAGGAACAAACCAT		PCR verification
	R	GAAGATGACGTAGGCCAGGT		
SimB TINF00247	U1	TCCCCCGGGTCTGGCAATGGTTGTCTATCTG	*Sma* I	Gene deletion
	U2	CGGGATCCAGAGCTGCTTCTTTGCCACA	*BamH* I	
	L1	GACTAGTGGGCTCGAGGGACCTTATAG	*Spe* I	
	L2	CGAGCTCCTGTCACTCCCAAGGGAAAA	*Sac* I	
	F	CCACGCTCAATGATGATGTC		PCR verification
	R	CCGGGACCAAAGTTCTCTATC		
SimC TINF00586	U1	GGAATTCGACCAAAAAATTCGGGTTGAA	*EcoR* I	Gene deletion
	U2	GGAATTCGGATTGGAAGGGGTAAAACG	*EcoR* I	
	L1	GCTCTAGAGTTGCTGTTGCGATTCCTTT	*Xba* I	
	L2	GCTCTAGACGGTATCCTTTCGACGACAT	*Xba* I	
	F	TCCTTGGCCCTCGTTTTACC		PCR verification
	R	GATGCGGTGTAGGTAGGAGC		
SimD TINF00536	U1	GGAATTCAGATGTCAGCGTCACGACAG	*EcoR* I	Gene deletion
	U2	TCCCCCGGGATCGCCATCATCTTCTCCAT	*Sma* I	
	L1	GCTCTAGAGATTAGGACGGAGCTGGTGA	*Xba* I	
	L2	GCTCTAGACCCCTACACGCTCATTTCAT	*Xba* I	
	F	GACCCATACAGAAGCCCAGA		PCR verification
	R	GCCTTCGTCTACAGCTGGTC		
SimE TINF00426	U1	CCGCTCGAGCGAAAAGAAGATGGGGACTG	Xho I	Gene deletion
	U2	CGGGATCCCCTTGGGCGAAACTGTAGAC	*BamH* I	
	L1	GACTAGTTTCTACACTTGCCCCTGGAC	*Spe* I	
	L2	CGAGCTCGAAGATGACCCGAAGATCCA	*Sac* I	
	F	CGCATGTGCGAGTATGTCTT		PCR verification
	R	CTCAGACACCTCACCAGCAC		
SimG TINF00267	U1	GGAATTCCTCGCCTACCATGGAGATGT	*EcoR* I	Gene deletion
	U2	AACTGCAGCTGGAACATCGTCCCAGACT	*Pst* I	
	L1	GGACTAGTAGCTTCAGCGTGTCCAAGAT	*Spe* I	
	L2	GCTCTAGATCTGCGATATCCCCAAGAAC	*Xba* I	
	F	CGTGACGTTGCTCTGACTGT		PCR verification
	R	CAAATCTTGGACACGCTGAA		
SimI TINF00470	U1	GGAATTCCACTCGGAAGCATTCTGACA	*EcoR* I	Gene deletion
	U2	TCCCCCGGGCAGCCACTCGTTCCCTACTC	*Sma* I	
	L1	GCTCTAGATCTGCAACAGTGCAAAGACC	*Xba* I	

	L2	CGAGCTCCCTCTCAAGCCTAGCCAATG	*Sac* I	
	F	GCCGTTTCCCTGAATGAGTA		PCR verification
	R	GCAGCAGATTTCGGAAAGAC		
SimJ TINF00351	U1	GGAATTCGGGAAGAAGCTCAACTCGTG	*EcoR* I	Gene deletion
	U2	CGGGATCCAAGTCTGATGGCTCCGAGAA	*BamH* I	
	L1	GACTAGTGGATATGCCGCTTATGGAGA	*Spe* I	
	L2	CGAGCTCTAAGAGCAGCCAAACACACG	*Sac* I	
	F	GCAAGTGACTCACCTCGTCA		PCR verification
	R	CGAGACGAACCCAAACATCT		
SimL TINF00394	U1	CCGCTCGAGTTCGAGTTTTCTCGGTCTTTTC	Xho I	Gene deletion
	U2	CGGGATCCCATGGCGTTAGGTCACACAC	*BamH* I	
	L1	GCTCTAGATATGTCGGCCCTCAGATACC	*Xba* I	
	L2	GCTCTAGAATGTTTGCTGCTTCAACGTG	*Xba* I	
	F	GTAACCAGATGGAGGCTTCG		PCR verification
	R	ATATGGCGTATTCGGTCCTG		
TINF06009	U1	GAATTCCTGCAGCCCATATAAATGCGCCTGCCAAC	*BamH* I	Fusion PCR for double gene deletion
	U2	TAATTGCGCGGATCCTCCTGACGTGACAAACAAGC		
	L1	ACCAGCCCTGACTAGTTTTGCTCGCACTTTGACAC	*Spe* I	
	L2	TAGATCTAGAACTAGTCGATACTGCTCGAGTGACG		
	F	TCCTGTACCCTGCTAGATGATG		PCR verification
	R	CTCCTGGGGCCCAACTAC		
SimL (WT*::SimL*)	gpdA-F	CCACCGCGGTGGAGCTCCTGATAAGCAGCACCACGAA	*Sac* I	Fusion PCR for gene overexpression in WT
	gpdA-R	GACGGCGGGGTGACGGTGGT		
	394-F	CGTCACCCCGCCGTCATGTCGTTCAAGAGGCCTTG		
	394-R	CAAAAGCTGGAGCTCTCAGGCCGTTCTAAAGTTGC		
	F	CTCAACAACACCACCGTCAC		PCR verification
	R	TCAGGCCGTTCTAAAGTTGC		
β-tubulin	Tub-F	TGTCTACAACGGCACCTCTG		RT-PCR reference
	Tub-R	AGGTCTCGTCCGAGTTCTCA		
SimA-SimN	SimA-F	CTGGCAGAGGAACAAACCAT		Primer pairs for RT-PCR analysis of gene expression in WT*::SimL*
	SimA-R	GAAGATGACGTAGGCCAGGT		
	SimB-F	CCACGCTCAATGATGATGTC		
	SimB-R	CCGGGACCAAAGTTCTCTATC		
	SimC-F	GTCTCTGTCTCTCCTCCGC		
	SimC-R	GCCGTCTTTCATAACAGGGC		
	SimD-F	GACCCATACAGAAGCCCAGA		
	SimD-R	GCCTTCGTCTACAGCTGGTC		
	SimE-F	AAGGGAGAGGAGGAAAAGGC		
	SimE-R	AACTGTTCCGTCACCTCCAT		
	SimG-F	AGCCTCTCACCTTCAATGCT		
	SimG-R	GACTTTGCAGCAACTCACCA		
	SimI-F	CCCTGCCAGCAAGATTTGTT		
	SimI-R	CGTAACGCTTCATCCATGCA		
	SimJ-F	GCAAGTGACTCACCTCGTCA		
	SimJ-R	CGAGACGAACCCAAACATCT		
	SimL-F	CCAACACCGAGGTCAGAGAT		
	SimL-R	ATGTTTGCTGCTTCAACGTG		
	SimN-F	GAGCCAGTCAGTCTTGGTGTC		
	SimN-R	CGGAGAGGTGATGGATGG		

* The introduced enzyme restriction sites are underlined.
